# Quality Assessment of Periapical Radiographs Taken by Dental Assistants Using the Recent Faculty of General Dental Practice (FGDP) Guidelines

**DOI:** 10.7759/cureus.68508

**Published:** 2024-09-03

**Authors:** Saqib Naeem Siddique, Malik Adeel Anwar, Hira Zaman, Irsam Haider, Aiman Ahmad, Muhammad Umair, Moghees Ahmed Baig

**Affiliations:** 1 Department of Operative and Pediatric Dentistry, University College of Dentistry, The University of Lahore, Lahore, PAK; 2 Department of Biomedical Engineering, Binghamton University, New York City, USA; 3 Department of Oral Pathology and Oral Diagnostics, University College of Dentistry, The University of Lahore, Lahore, PAK; 4 Department of Oral and Maxillofacial Surgery, University College of Dentistry, The University of Lahore, Lahore, PAK

**Keywords:** diagnostically acceptable, faculty of general dental practice guidelines, quality assessment, dental assistants, periapical radiographs

## Abstract

Background: Periapical radiographs play a pivotal role in dentistry, offering invaluable insights essential for various dental procedures.

Objective: This study aims to systematically assess the quality of intraoral periapical (IOPA) radiographs evaluating adherence to the recent guidelines established by the Faculty of General Dental Practice (FGDP).

Methods: A cross-sectional study was conducted at the University College of Dentistry (UCD), employing a non-probability consecutive sampling technique to acquire a calculated sample of 300 IOPA radiographs from the operative, oral surgery, and oral radiology departments. Two senior faculty members evaluated the radiographs according to the recent two-tier grading system outlined in the FGDP guidelines.

Results: The study revealed that 197 (65.67%) of the assessed radiographs were diagnostically acceptable, while 103 (34.33%) were deemed diagnostically unacceptable. Contrast problems emerged as the most prevalent issue, accounting for 85 (28.3%) of the cases. Other common problems included incorrect film positioning in 66 (22%), incorrect vertical cone angulation in 37 (12.3%), incorrect horizontal cone angulation in 11 (3.7%), and incorrect processing in 15 (5%) of the IOPA radiographs.

Conclusion: This study revealed that approximately two-thirds of the IOPA radiographs were deemed diagnostically acceptable. However, contrast issues emerged as the predominant concern affecting image quality. These findings highlight the critical importance of continuous quality improvement initiatives in radiographic practices to enhance diagnostic precision and ensure optimal patient care.

## Introduction

Radiographs are indispensable tools for diagnosing and assessing various dental disorders and procedures, both intra and post-operatively [1]. Intraoral periapical (IOPA) radiographs provide detailed information about teeth and their supporting structures, commonly used for identifying periapical diseases, crown and root fractures, pulp and root canal morphologies, with its numerous variations across different teeth, dental anomalies related to cleft lip and palate, and the assessment of the health of the supporting alveolar bone. [2-4]. In endodontic treatment, periapical radiographs play a crucial role in both diagnosing pathology and planning treatment [5].

Accurate interpretation of radiographs depends on obtaining high-quality images free from processing errors and technical flaws [5]. Poor image quality can significantly compromise the accuracy of diagnosis [6,7]. Previous research has documented various types and frequencies of errors occurring during radiographic procedures which mainly include image acquisition and processing. Regardless of the technique employed - whether bisecting or paralleling technique - the most common errors relate to film positioning, radiation beam orientation, radiographic contrast (light or dark image), vertical or horizontal angulations, and processing errors such as stains, streaks, or inadequate use of fixative [5,8,9].

Ionizing radiation poses numerous hazards for both patients and dental personnel involved in radiographic inspections [1]. Ionizing radiation poses numerous hazards for both patients and dental personnel involved in radiographic inspections. Although the dose of radiation from a single IOPA radiograph is relatively low, repeated exposures can accumulate, especially when patients undergo unnecessary radiation exposure due to retakes resulting from inadequate diagnostic quality, thereby increasing the risk of radiation-related health issues [10-12]. Tracking the types and frequency of errors in radiographic practices is essential for quality evaluation, enabling the detection and resolution of technical flaws to minimize repetitions and patient radiation exposure [5,13].

Accurate radiographic interpretation relies on several factors, including the availability of fundamental information, high-quality images, and the absence of technical and processing flaws. The literature extensively documents quality audits addressing concerns such as radiographic image quality, processing, and documentation [14-16]. Effective quality assurance measures are essential for good dental practice [17]. Knowing when to prescribe radiographs, understanding the type of radiographs needed, and interpreting them correctly are vital to ensuring timely and accurate diagnoses [18].

Dose optimization, dose limitation, and justification constitute the fundamental principles of radiation protection. The prime objective of the radiographic examination is to achieve diagnostic efficacy while minimizing radiation exposure to patients and healthcare personnel, adhering to the principle of “As Low as Reasonably Practicable” [17,19].

Dental assistants, trained individuals providing chairside patient care, often perform radiographs in institutional settings, including before, during, and after dental procedures, particularly endodontic interventions, to assess treatment outcomes [1].

Previously, radiograph quality was assessed using a three-grade system - excellent, diagnostically acceptable, and unacceptable - based on guidance from the National Radiological Protection Board (NRPB). However, in October 2020, this classification system was replaced by a simplified two-grade system introduced by the Faculty of General Dental Practice (FGDP), which categorizes radiographs as either diagnostically acceptable or not acceptable [20]. The objective of this study was to evaluate the quality of IOPA radiographs taken by dental assistants according to the recent guidelines established by the FGDP.

## Materials and methods

The study received ethical approval from the Institutional Review Board of the University College of Dentistry (UCD) (ERC No UCD/ERCA/530). A cross-sectional study design was employed, utilizing non-probability consecutive sampling to collect 300 IOPA radiographs taken by dental assistants over three months from three departments at UCD: Operative Dentistry, Oral Surgery, and Oral Diagnostics. The sample size of 300 radiographs is calculated with a 90% confidence level, 4.5% absolute precision, and by taking the expected percentage of diagnostically acceptable Periapical Radiographs as 45% [1].

Radiographs were captured using the DIGORA Optime system (type DXR-60-01, manufactured in Finland). Only the most recent 300 IOPA radiographs were included in this study to ensure a focused assessment of current radiographic practices. All other types of radiographs, such as panoramic, bitewing, lateral cephalometric, or cone beam computed tomography images, were excluded from the analysis to maintain consistency and specificity in evaluating IOPA quality. Additionally, radiographs from pediatric patients, specifically those under six years old, were also excluded to eliminate potential variations in anatomical considerations and developmental factors that may affect image quality and interpretation. The assessment was conducted by two senior faculty members using a DELL Latitude 3510, 15.6-inch LED display. Inter-examiner reliability, with a score of 0.804 indicating fair agreement, was established.

Various errors were evaluated in the radiographs, including incorrect film positioning (identified by crown or root cut presence), faulty vertical and horizontal cone angulations, incorrect contrast, and processing errors (streaks, scratch, exposure to white light of the sensor plate). Radiograph quality was assessed according to the most recent guidelines from the FGDP categorizing images as either diagnostically acceptable “A” or not acceptable “N”. FGDP requires at least 90% of radiographs to be diagnostically acceptable for conventional film imaging while for digital imaging, these thresholds were set at 95%. As digital imaging was used at UCD, radiographs were assessed accordingly (Table 1) [20].

**Table 1:** Subjective Image Quality Ratings of Dental Radiographs and CBCT Images by FGDP. CBCT: cone beam computed tomography; FGDP: Faculty of General Dental Practice

Quality Rating	Basis	Target (Percentage of Radiographs or CBCT Images in Sample)	
		Digital imaging	Film imaging
Diagnostically acceptable (“A”)	No errors or minimal errors in either patient preparation exposure, positioning, image (receptor) processing, or image reconstruction and of sufficient image quality to answer the clinical question	Not less than 95%	Not less than 90%
Diagnostically not acceptable (“N”)	Errors in either patient preparation, exposure, positioning, image (receptor) processing, or image than 5% than 10% reconstruction which render the image diagnostically unacceptable	Not less than 95%	Not less than 90%

Data analysis was performed using the Statistical Package for the Social Sciences (SPSS version 25). Frequencies and percentages were used to present qualitative variables. A chi-square test was applied to assess the association between various errors in radiographs and their diagnostic acceptability, with a statistically significant level set at p ≤ 0.05.

## Results

**Figure 1:**
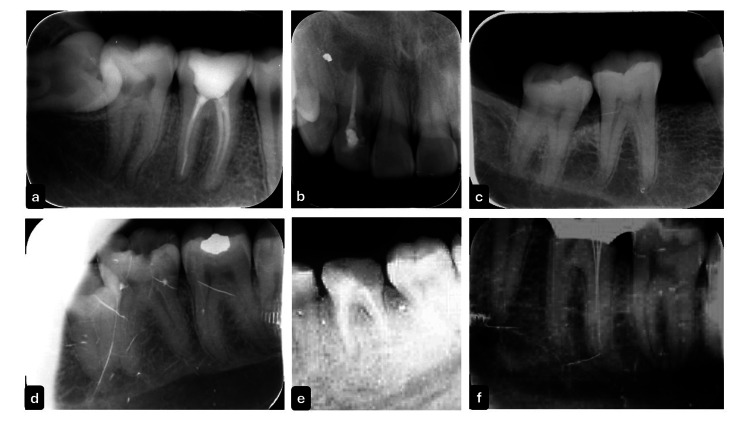
Diagnostically Acceptable and Unacceptable IOPA Radiographs. a-c) Diagnostically acceptable images demonstrating proper contrast, positioning, angulation and processing, d-f) Diagnostically unacceptable images due to issues such as poor positioning, incorrect contrast and improper angulation. IOPA: intraoral periapical

A total of 300 IOPA radiographs were evaluated, with 197 (65.67%) deemed diagnostically acceptable and 103 (34.33%) considered diagnostically unacceptable (Figure 1).

Analysis of individual defects revealed that contrast problems were the primary reason for radiographs being diagnostically unacceptable, accounting for 85 (28.3%) of cases, followed by incorrect film positioning among 66 (22.0%) and incorrect vertical cone angulation 37 (12.30%) of the radiographs graded. Incorrect radiograph processing was observed in 15 (5%) of the radiographs, while horizontal cone angulation issues were reported in only 3.70%. Table 2 provides a detailed breakdown of these individual defects.

**Table 2:** Distribution of Radiographic Outcomes and Associated Defects.

Category	Number (n)	Percentage (%)
Diagnostically Acceptable Radiographs	197	65.67
Diagnostically Unacceptable Radiographs	103	34.33
Individual Defects		
Incorrect Contrast	85	28.30
Incorrect Film Positioning	66	22.00
Incorrect Vertical Cone Angulation	37	12.30
Incorrect Horizontal Cone Angulation	11	3.70
Incorrect Radiograph Processing	15	5.00

Defective contrast, incorrect film orientation, and incorrect vertical and horizontal cone angulations emerged as statistically significant factors impacting radiographic quality in the study. The results of statistical analysis revealed significant associations between these defects and the overall diagnostic acceptability of the radiographs.

Specifically, incorrect vertical cone angulation was observed in seven (3.6%) of acceptable radiographs compared to 30 (29.1%) of unacceptable ones (p < 0.001). Similarly, incorrect horizontal cone angulation occurred in four (2.0%) of acceptable radiographs versus seven (6.8%) of unacceptable ones (p = 0.037). Incorrect film positioning was found in 9.1% of acceptable radiographs compared to 46.6% of unacceptable ones (p < 0.001). Moreover, defective contrast was present in 31 (15.7%) of acceptable radiographs and 54 (52.4%) of unacceptable radiographs (p < 0.001). The results are summarized in Table 3.

**Table 3:** Analysis of Radiographic Quality and Individual Defects. *Significant p-value

Defect	Presence of Defect	Acceptable (A)		Not Acceptable (N)		p-value
		n	%	n	%	
Incorrect contrast	Yes	31	15.7%	54	52.4%	<0.001*
	No	166	84.3%	49	47.6%	
	Total	197	100%	103	100%	
Incorrect film positioning	Yes	18	9.1%	48	46.6%	<0.001*
	No	179	90.9%	55	53.4%	
	Total	197	100%	103	100%	
Incorrect vertical cone angulation	Yes	7	3.6%	30	29.1%	<0.001*
	No	190	96.4%	73	70.9%	
	Total	197	100%	103	100%	
Incorrect horizontal cone angulation	Yes	4	2.0%	7	6.8%	0.037*
	No	193	98.0%	96	93.2%	
	Total	197	100%	103	100%	
Incorrect Radiograph Processing	Yes	8	4.06%	7	6.7%	0.303
	No	189	95.9%	96	93.2%	
	Total	197	100%	103	100%	

## Discussion

Quality assurance programs in dental radiography are essential and must include thorough monitoring of radiographic image quality [6]. Frequent quality control checks provide a reliable method to ensure dental team members meet the expected benchmarks, enhancing clinical practices by minimizing the need for repeat imaging, especially in cases of complex oro-facial injuries from roadside accidents. In such scenarios, accurate imaging is critical for proper diagnosis and treatment planning, and repeating radiographs is undesirable, as the patient is often in a compromised condition [1,21,22]. In this study, we aimed to categorize IOPA taken at UCD according to the recent guidelines set by the FGDP. The radiographs were classified as either diagnostically acceptable (“A”) or diagnostically unacceptable (“N”), and we calculated the percentages of various faults found.

In this study, we utilized the latest FGDP criteria for grading radiographs, categorizing them as either diagnostically acceptable or unacceptable [20], without any further subcategories. This approach contrasts with other studies that employed the previous three-tier NRPB grading system, which included additional classifications: excellent (grade 1), diagnostically acceptable (grade 2), and unacceptable (grade 3) which was withdrawn in 2020 [23]. We were unable to identify any similar criteria specifically designed for assessing the quality of radiographic errors.

Our findings revealed that the most common error was incorrect contrast (28.30%), notably higher than the 12% reported by Ali et al., 7.9% by Javed et al., and 7% by Patankar et al. [1,17,6]. This higher incidence might be due to the dental assistants' challenges in properly adjusting the machine’s contrast or inaccuracies in computer settings. Film positioning errors were observed in 22% of our radiographs, consistent with Elangovan et al. (22.2%) but lower than the higher rates reported by Javed et al. (49.3%) and Patankar et al. (54.3%) [15,17,6]. These variations might be attributed to differences in the training levels of dental assistants.

Incorrect vertical angulation was observed in 12.30% and horizontal angulation in 3.70% of our radiographs, both better than the rates reported by Javed et al. (37.7% vertical and horizontal angulation combined), Elangovan et al. (24.2% and 13.3%), and Patankar et al. (25.6% and 24.8%), but higher than Ali et al. (4% and 2%) [15, 3,4,1]. We observed a 5% rate of incorrect radiograph processing, better than Elangovan et al. (11.5%), Ali et al. (11%), and Patankar et al. (10.1%), combined developer and fixer issues) [15,1,6]. These discrepancies may stem from variations in the radiographic protocols and the experience level of the dental assistants involved in each study. Table 4 summarizes the percentage of various defects found in our study compared to other studies, highlighting the differences in performance across different parameters.

**Table 4:** Comparison of Radiographic Defects Across Studies.

Studies/Defects	Incorrect				
	Contrast	Film Positioning	Vertical Angulation	Horizontal Angulation	Radiograph Processing
Our study	28.30%	22%	12.30%	3.70%	5%
Ali et al. [1]	12%	14%	4%	2%	11%
Patankar et al. [6]	7%	54.30%	25.60%	24.8%	10.1%
Elangovan et al. [15]	-	22.2%	24.20%	13.30%	11.2%
Javed et al. [17]	7.90%	49.30%	37.7%		-

Our study using the recent FGDP guidelines found that 65.67% of the radiographs were diagnostically acceptable, falling short of the FGDP target of 95% acceptable radiographs. However, the study conducted by Nalawade et al. using these recent two-grade system FGDP guidelines had 70% of radiographs deemed diagnostically acceptable taken by dental assistants which was quite closer to what we found in our study [24].

However, other studies employed the previous NRPB grading system, which included categories such as excellent (grade 1), diagnostically acceptable (grade 2), and unacceptable (grade 3). Since we found only one study from Oman [24] that utilized the recent two-grade system provided by FGDP, we combined the excellent and diagnostically acceptable radiographs (grades 1 and 2) from the studies using the NRPB grading system as diagnostically acceptable for comparison with our results.

According to this system, grade 1 radiographs should constitute at least 70%, while grade 2 should not fall below 20%, totaling 90% for acceptable radiographs. When the old grading system was applied, Patankar et al. got 57.4%, Ali et al. reported 77%, Javed et al. achieved 83.3%, and Khan et al. found 84.02% acceptable radiographs, all significantly better than our results [6,1,17,25]. Overall, our study’s percentages of acceptable radiographs (65.67%) did not meet the targets set by the recent FGDP guidelines. Table 5 presents a comparison of the percentages of diagnostically acceptable and unacceptable radiographs between our study and those conducted using the NRPB grading system.

**Table 5:** Comparison of Diagnostically Acceptable and Unacceptable Radiographs: Current vs Other Studies. *Studies used National Radiological Protection Board (NRPB) guidelines; Grade 1 and Grade 2 categories were combined into “diagnostically acceptable” for comparison.

Studies/Categories	Diagnostically Acceptable Radiographs (A)	Diagnostically Unacceptable Radiographs (N)
Our study	65.67%	34.33%
Nalawade et al. [24]	70%	30%
*Ali et at. [1]	77%	33%
*Patankar et al. [6]	57.40%	42.60%
*Javed et al. [17]	83.30%	16.70%
*Khan et al. [25]	84.02%	15.98%

However, it is important to acknowledge certain limitations. First, the examiners were not trained radiologist assistants, which might have led to some errors being overlooked. Second, all the radiographs were taken using the bisecting angle technique, excluding those taken with the paralleling technique. Ensuring the quality of radiographs is crucial for accurate diagnosis, minimizing patient radiation exposure, and enhancing treatment planning. To further improve radiograph quality, several steps can be taken. For instance, the introduction of position-indicating devices can help achieve better X-ray accuracy. Additionally, training in radiographic techniques is essential. Regular audits can also help maintain high standards in radiograph quality. Periodic and thorough audits are crucial to finding and fixing common mistakes, making sure we continuously improve the quality of our radiographs.

## Conclusions

This study revealed that only two-thirds of the IOPA radiographs evaluated were considered diagnostically acceptable, highlighting a significant quality gap primarily due to contrast issues. These findings emphasize the critical need for continuous quality improvement initiatives in dental radiography to enhance diagnostic accuracy and patient safety. The results serve as a call to action for dental practices to prioritize high-quality imaging standards, ensuring that diagnostic radiographs meet established guidelines and ultimately contributing to improved patient care and treatment outcomes.
